# Acalculous cholecystitis is a common extrahepatic manifestation of hepatitis E and suggests a more serious condition

**DOI:** 10.1186/s12985-023-02045-8

**Published:** 2023-04-24

**Authors:** Xuemei Cao, Wei Jiang, Lingfeng Shi, Yanping Wang, Jie Chen, Wenxiang Huang, Shujun Zhang

**Affiliations:** 1 Chongqing Key Laboratory of Infectious Diseases and Parasitic Diseases, Department of Infectious Diseases, The First Affiliated Hospital of Chongqing Medical University, No. 1 Youyi Road, Yuzhong District, Chongqing, 400016 China; 2 Department of Infectious Diseases, Youyang Hospital, A Branch of The First Affiliated Hospital of Chongqing Medical University, Chongqing, China; 3 Department of Laboratory Medicine, The First Affiliated Hospital of Chongqing Medical University, Chongqing, China

**Keywords:** Hepatitis E, Acalculous cholecystitis, Spontaneous peritonitis, Hospital stay

## Abstract

**Background:**

This study aimed to understand the incidence and clinical significance of acalculous cholecystitis in patients with acute hepatitis E (HE).

**Patients and methods:**

A single center enrolled 114 patients with acute HE. All patients underwent imaging of the gallbladder, and patients with gallstones and cholecystectomy were excluded.

**Results:**

Acalculous cholecystitis was found in 66 patients (57.89%) with acute HE. The incidence in males was 63.95%, which was significantly higher than in females (39.29%) (*P* = 0.022). The mean length of hospital stay and the incidence of spontaneous peritonitis in patients with cholecystitis (20.12 ± 9.43 days and 9.09%, respectively) were significantly higher than those in patients without cholecystitis (12.98 ± 7.26 days and 0%, respectively) (*P* < 0.001 and *P* = 0.032). Albumin, total bile acid, bilirubin, cholinesterase, and prothrombin activity in patients with cholecystitis were significantly inferior to those in patients without cholecystitis (*P* < 0.001, *P* < 0.001, *P* < 0.001, *P* < 0.001 and *P* = 0.003, respectively). After correction by multivariate analysis, albumin and total bile acid were found to be closely related to acalculous cholecystitis in HE.

**Conclusion:**

Acalculous cholecystitis is very common in patients with acute HE, and may serve as a predictor of increased peritonitis, synthetic decompensation, and longer hospital stay.

## Introduction

Hepatitis E (HE) is a disease mediated by the hepatitis E virus (HEV) that is transmitted mainly through the digestive tract. Most people infected with HEV are asymptomatic or self-limiting, with a mortality rate of up to 3% in young people [1] and 30% in pregnant women [2]. HE virions are quasi-enveloped or non-enveloped, 27–34 nm in diameter, with a single capsid protein, which belongs to the species *Paslahepevirus balayani,* genus *Paslahepevirus* and family *Hepeviridae* [3], and contains four open reading frames (ORFs). ORF1 encodes non-structural proteins for replication, including methyltransferase, ribonucleic acid (RNA) helicase, RNA polymerase and papain-like cysteine protease. ORF2 encodes the capsid protein, that is used for vaccine preparation. ORF3 partially overlaps with ORF2 and encodes a multifunctional protein that may be involved in viral secretion [4]. Recently, a new ORF4 was discovered in genotype 1, which may mediate the interaction of the virus with the host protein to participate in its replication [5]. To date, eight genotypes have been identified [6, 7]. Genotypes 1 and 2 infect only humans and are transmitted primarily in developing countries through the fecal–oral route via contaminated water and food [8]. Genotypes 3 and 4 can infect pigs, deer, and other zoonoses and can be transmitted through contaminated water and food, contact with infected animals, and transfusions of contaminated blood products [9]. Genotypes 5 and 6 have been found in wild animals in Japan. Genotypes 7 and 8 have been detected in camels from the Middle East and China, respectively.

Acalculous cholecystitis refers to inflammation of the gallbladder without gallstones, which is usually caused by mechanical factors, chemical materials, or infection [10]. Acalculous acute cholecystitis is identified in approximately 5–10% of patients with acute cholecystitis. Unlike calculous cholecystitis, acalculous cholecystitis is the most frequent complication in critically ill patients, with an incidence ranging from 0.5 to 18%. Acalculous cholecystitis can occur in conjunction with multiple organ failure, and its occurrence often indicates multisystemic failure [11]. Although it was reported in 1987 that acalculous cholecystitis may be an extrahepatic complication of liver disease [12], viral hepatitis-related acalculous cholecystitis is mostly reported in relation to hepatitis A virus (HAV) [13–20] with few reports in hepatitis B virus (HBV) [21] and C [22–24]. Metabolites of the virus may invade the wall of the gallbladder or biliary epithelial cells, leading to cholestasis, which in turn results in acalculous cholecystitis [10, 25]. The extrahepatic manifestations of HE reported to date mainly include acute pancreatitis [26], neurological diseases [27, 28], kidney injury [29], and hematologic disorders [30, 31]. HE-related cholecystitis was not reported in a study from Qatar until 2009; however, there were only two cases [32]. Subsequent reports of HE related cholecystitis were also case reports [25], two of which were coinfected with HAV [33] and *Salmonella typhi* [34] making it difficult to reveal the significance and mechanism of cholecystitis in HE.

Therefore, we retrospectively investigated 114 patients diagnosed with acute HE to demonstrate the significance of acalculous cholecystitis in acute HE.

## Patients and methods

### Patients and definition of acute hepatitis E and acute acalculous cholecystitis

Since no patients with chronic HE was identified in our hospital, only patient with acute HE was included in this study. Acute HE is defined as markedly elevated transaminases (alanine transaminase (ALT) ≥ 2.5 × ULN) with positive anti-HEV immunoglobulin M (IgM) or HEV RNA. A total of 127 patients diagnosed with sporadic acute HE at the First Affiliated Hospital of Chongqing Medical University between January 2013 and April 2022 were included in the initial screening. Of the 127 patients, seven were excluded due to cholecystectomy, three were excluded due to gallstones, two were excluded due to imaging examination of the undiagnosed gallbladder, and one was excluded due to unclear gallbladder display. Ultimately, 114 patients were included in the analysis. Cholecystitis was defined as edema of the gallbladder wall on ultrasound, computed tomography or magnetic resonance imaging with a thickness of > 3 mm [35, 36].

### Detection of anti-HEV IgM and IgG

Serum anti-HEV-IgM and anti-HEV-IgG antibodies were detected using enzyme-linked immunosorbent assay kits (Beijing Hyundai Gundam from 2012 to 2019, and Beijing Wantai Company from 2019 to 2022). A S/CO value ≥ 1 was considered positive, and all positive results were confirmed by re-examination.

### RNA extraction, sequencing, and phylogenetic analysis

Viral RNA was extracted from 200 µL of serum samples using Trizol LS reagent (Invitrogen). Reverse transcription of the extracted RNA was carried out in a 20 µl reaction mixture containing 20 U of RNA sin (Takara), 1 × RT buffer (Takara), 1 mM each dNTP (Takara), 5 U of AMV reverse transcriptase (Takara), and 2.5 uM of reverse transcription primer E5:5′-ctacacgaaaccgaragw-3′ (r = a OR g, w = a OR c). The mixture was incubated at room temperature for 5 min, then at 42 °C for 60 min and at 95 °C for 5 min. Then,2 µl of the obtained cDNA was added to a 20 µl reaction mixture containing 0.5 mM each of the primers E5 and E1:5′-ctgtttaaycttgctgacac-3′ (y = c OR t), 1 U of Taq DNA polymerase (Takara), and 10 × PCR buffer (Takara), overlaid with 20ul of mineral oil, and subjected to 35 cycles of PCR in a thermos-cycler (94 °C, 40 s; 53 °C, 40 s; 72 °C, 40 s). Then, 2 µl of the first-round PCR product was amplified for a further 25 cycles (94 °C, 40 s; 53 °C, 40 s; 72 °C, 40 s) using the internal primers E2:5′-gacagaattgatttcgtcg-3′) and E4:5′-gtcctaatactrttggttgt-3′ (r = an OR g). The length of the product corresponding to the ORF2 sequence was 189 bp (6298nt–6486nt). All the PCR products were subjected to bidirectional sequencing (Invitrogen). The phylogenetic tree was constructed using MEGA 11 software using the neighbor-joining method based on the reference sequences from genotype 1 to genotype 8, as recommended by Smith et al. [6]. The HEV genotyping tool (https://www.rivm.nl/mpf/ typingtool/hev/, https://www.genome-detective.com /app/typing tool/virus/) was also used for genotype confirmation and further subtype differentiation. The amplified sequences were deposited in GenBank under accession no. OP974689, OP974690, OP999127, OQ054343-OQ054355.

### Statistics

Quantitative data are presented as the mean ± standard deviation and were compared using Student’s t test. The Chi-square test or Fisher’s exact test was used to enumerate the data. The odds ratios (OR) for all variables were calculated using univariate and multivariate logistic regression. All tests were two-tailed, and *P* values of < 0.05 were considered significant.

## Results

### Demographic characteristics

**Fig. 1:**
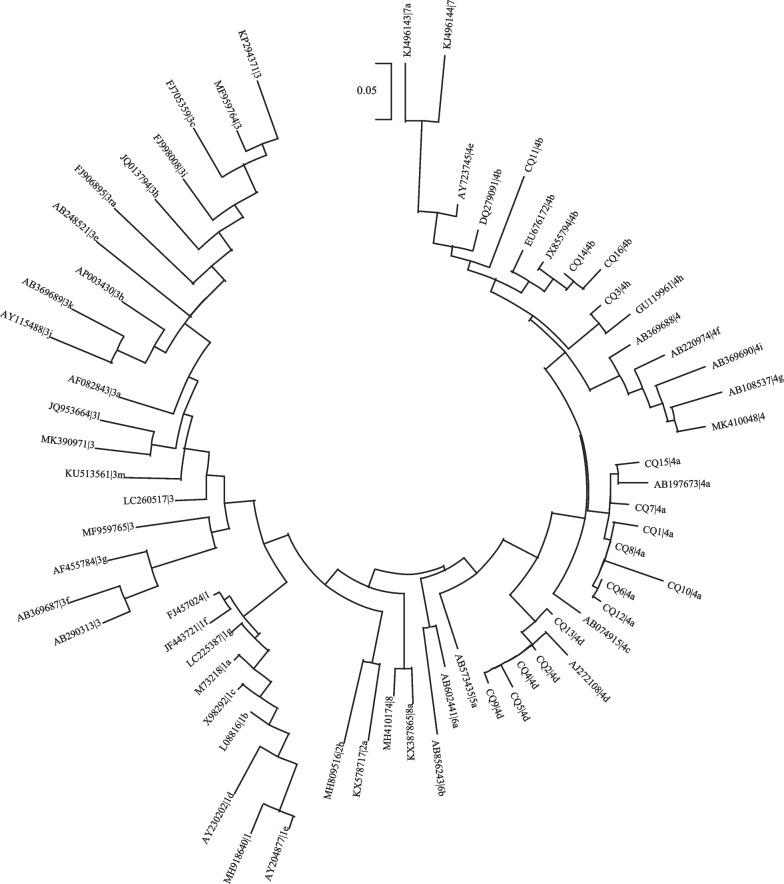
Phylogenetic analysis of fragments from 16 patients positive for HEV RNA

Of the 114 patients, 86 were males (75.44%) and 28 were females (24.56%). One of the women was pregnant at the time of diagnosis. The male-to-female ratio was 3.07:1. The ages of the patients ranged from 15 to 90 years (52.35 ± 14.86 years), and 36 patients (31.58%) were over 60 years old. Eight patients had liver cirrhosis, including five with alcoholic liver cirrhosis, two with hepatitis B cirrhosis, and one with an unknown cause. Among the 15 patients coinfected with other hepatitis viruses, 11 were coinfected with HBV, two with HAV, and two with HAV and HBV. Anti-HEV-IgM was positive in 112 patients (98.25%), anti-HEV-IgG was positive in 84 patients (73.68%), and both were positive in 82 patients (71.93%). Serum samples were obtained from 41 patients after January 2019, of whom 16 (39.02%) were positive for HEV RNA. CQ1, CQ6, CQ7, CQ8, CQ10, CQ12 and CQ15 were assigned to genotype 4a, CQ11, CQ14 and CQ16 to genotype 4b, CQ2, CQ4, CQ5, CQ9 and CQ13 to genotype 4d, and CQ3 to genotype 4 h (Fig. 1).

Samples were numbered from CQ1 to CQ16 and the reference sequences were represented by GenBank ID and genotype (separated by the vertical line).

### Incidence of acalculous cholecystitis in patients with acute HE

**Fig. 2:**
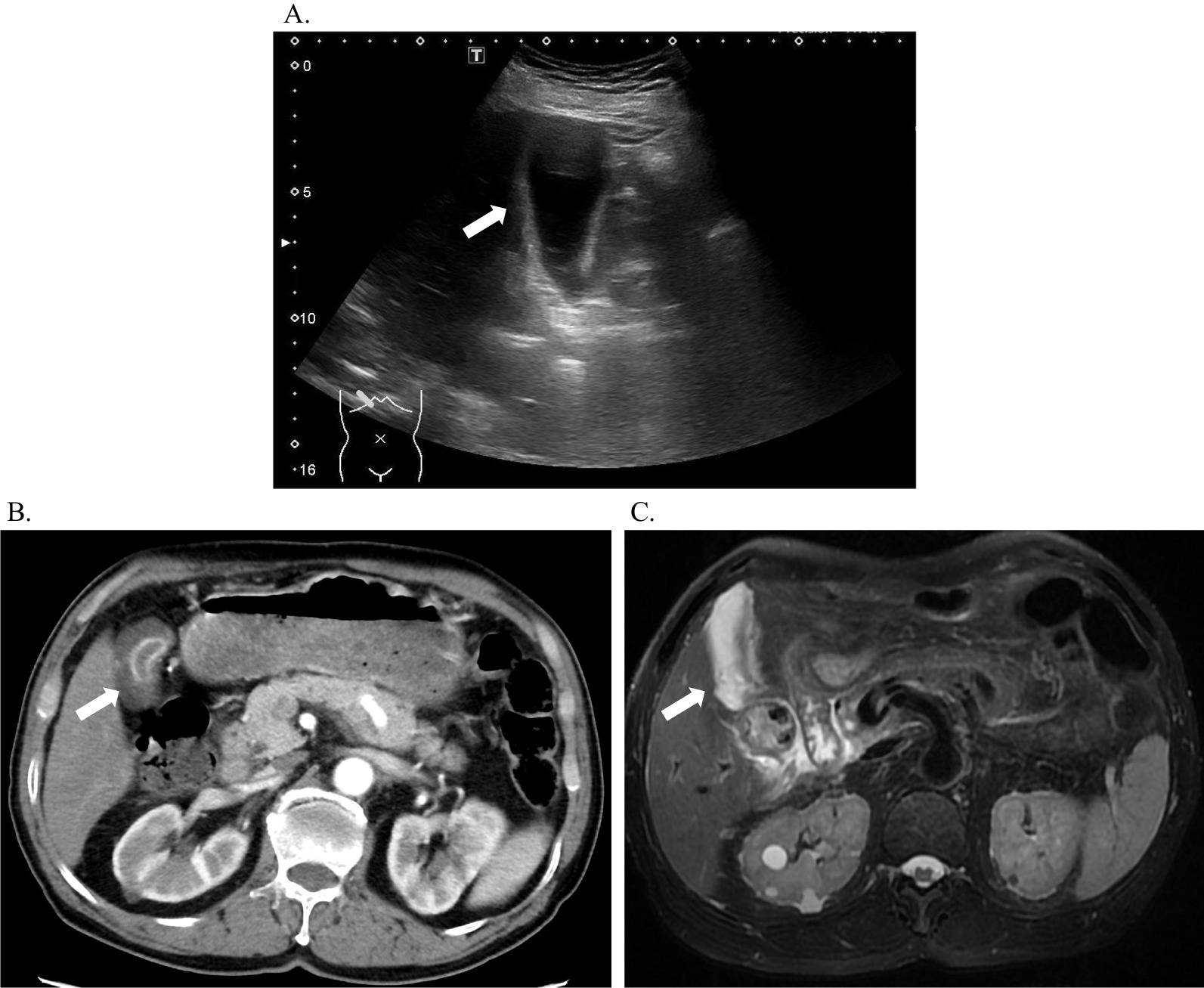
Images of gallbladder in two patients with acute hepatitis E. Abdominal ultrasound (**a**) showed significant thickening of the gallbladder wall (white arrow) in case 1. Both CT (**b**) and MRI (**c**) showed the presence of acalculous cholecystitis (white arrow) in case 2

Surprisingly, acute acalculous cholecystitis was found to be very common in patients with acute HE (Fig. 2), with a total of 66 positive cases, accounting for 57.89% (95% CI 48.79–66.99%). Furthermore, we grouped patients by sex, age, genotype (subtype), coinfection and presence of liver cirrhosis to compare the incidence of cholecystitis. As shown in Table 1, the incidence of cholecystitis in male patients was significantly higher in males than in females. There were also no significant differences in the incidence of cholecystitis between groups with different genotypes and superinfections. Although seven of the eight patients with liver cirrhosis developed cholecystitis, the incidence rate was as high as 87.5%, while only 55.66% of patients without liver cirrhosis had cholecystitis. However, the difference was not statistically significant possibly because of the small sample size of patients with underlying liver cirrhosis.

**Table 1:** Incidence of acalculous cholecystitis in various groups

Group	Incidence of cholecystitis	*P* value
Gender		
Male (n = 86)	55/86 (63.95%)	
Female (n = 28)	11/28 (39.29%)	0.022
HEV genotype		
4a (n = 7)	4/7 (57.14%)	
4b (n = 3)	1/3 (33.33%)	0.778
4d (n = 5)	2/5 (40.00%)	0.921
4 h (n = 1)	0/1 (00.00%)	0.408
Coinfection		
Without coinfection (n = 99)	58/99 (58.59%)	
Coinfected with HBV (n = 11)	6/11 (54.55%)	0.797
Coinfected with HAV (n = 2)	1/2 (50.00%)	0.807
Coinfected with HAV and HBV (n = 2)	1/2 (50.00%)	0.807
Liver cirrhosis		
With (n = 8)	7/8 (87.50%)	
Without (n = 106)	59/106 (55.66%)	0.079
Age		
< 60y (n = 78)	42/78 (53.85%)	
≥ 60y (n = 36)	24/36 (66.67%)	0.197

### Comparison between HE patients with or without acalculous cholecystitis

To further clarify the significance of cholecystitis in HE, we compared relevant parameters between the groups with and without cholecystitis. As shown in Table 2, acalculous cholecystitis was more common in male patients with acute HE. There was no significant difference in the incidence of liver failure or mortality between the two groups. However, the average hospital stay for HE patients with cholecystitis was nearly 20 days, which was significantly higher than the 12.98 days for patients without cholecystitis. In terms of laboratory indicators, those reflecting liver anabolism and reserve function, including albumin (ALB), total bile acid (TB), bilirubin, cholinesterase (CHE), and prothrombin activity (PTA), were significantly lower in the cholecystitis group than in the cholecystitis group.

**Table 2:** Comparison between the group of patients with and without cholecystitis

Parameters	With cholecystitis (n = 66)	Without cholecystitis (n = 48)	*P* value
Age (years)	54.14 ± 15.34	49.9 ± 13.97	0.133
Gender			
Male (%)	55 (83.33%)	31 (64.58%)	0.022
Female (%)	11 (16.67%)	17 (35.42%)	
Hospital days	20.12 ± 9.43	12.98 ± 7.26	< 0.001
Liver cirrhosis no. (%)	7 (10.61%)	1 (2.08%)	0.079
Liver failure no. (%)	12 (18.18%)	6 (12.50%)	0.411
Death no. (%)	5 (7.58%)	1 (2.08%)	0.195
Spontaneous peritonitis no. (%)	6 (9.09%)	0 (0%)	0.032
Upper gastrointestinal hemorrhage no. (%)	2 (3.03%)	1 (2.08%)	0.755
Hepatic encephalopathy no. (%)	1 (1.52%)	1 (2.08%)	0.820
Hepatorenal syndrome no. (%)	2 (3.03%)	0 (0%)	0.224
ALB (g/L)	30.58 ± 4.79	35.65 ± 4.27	< 0.001
TBA (µmol/L)	171.66 ± 141.74	83.37 ± 94.02	< 0.001
TB (µmol/L)	241.31 ± 136.67	102.07 ± 87.47	< 0.001
DB (µmol/L)	204.12 ± 122.47	82.54 ± 76.72	< 0.001
ALT (U/L)	1670.84 ± 1297.83	1614.60 ± 1716.71	0.842
AST (U/L)	1290.40 ± 1138.70	1151.55 ± 1536.80	0.580
ALP (U/L)	213.02 ± 79.89	194.20 ± 97.37	0.26
GGT (U/L)	268.60 ± 274.15	286.76 ± 245.50	0.716
LDH (U/L)	710.05 ± 716.46	613.07 ± 558.51	0.448
CHE (U/L)	3836.86 ± 1426.02	5674.67 ± 2088.57	< 0.001
BUN (µmol/L)	6.66 ± 6.19	5.17 ± 1.69	0.112
Cr (µmol/L)	80.78 ± 55.79	71.57 ± 24.13	0.291
PTA (%)	74.72 ± 24.51	88.87 ± 25.46	0.003
TC (mmol/L)	3.57 ± 1.11	4.43 ± 1.86	0.037
TG (mmol/L)	2.45 ± 1.37	1.69 ± 0.97	0.030
LDL (mmol/L)	1.45 ± 0.98	2.45 ± 1.29	0.002
WBC (× 10^12^/L)	5.29 ± 2.15	4.88 ± 1.61	0.270
N (%)	63.54 ± 10.59	58.19 ± 12.06	0.013

*ALB* albumin, *TBA* total bile acid, *TB* total bilirubin, *DB* direct bilirubin, *ALT* alanine aminotransferase, *AST* aspartate aminotransferase, *ALP* alkaline phosphatase, *GGT* gamma-glutamyltransferase, *LDH* lactate dehydrogenase, *CHE* cholinesterase, *BUN* blood urea nitrogen, *Cr* creatinine, *PTA* prothrombin activity, *TC* total cholesterol, *TG* triglyceride, *LDL* low density lipoprotein, *WBC* white blood cell, *N* neutrophils, *HBV* hepatitis b virus, *HAV* hepatitis a virus

### Risk factors of acalculous cholecystitis among patients with HE by univariate and multivariate analysis

To further identify the risk factors for acalculous cholecystitis in patients with HE, we first performed a univariate analysis of factors that may be associated with cholecystitis, and further performed a multivariate analysis if these factors were statistically significant. Complications including spontaneous peritonitis, upper gastrointestinal hemorrhage, hepatic encephalopathy and hepatorenal syndrome were excluded, because the P value of the Hosmer–Lemeshow test of these variables was less than 0.05 so regression analysis could not be performed. As shown in Table 3, only ALB and TB were closely associated with acalculous cholecystitis in patients with HE after correction using multivariate analysis.

**Table 3:** Univariate and multivariate logistic regression analysis of acalculous cholecystitis in patients with hepatitis E

Parameters	Univariate analysis			Multivariate analysis		
	OR	95% CI	*P* value	OR	95% CI	*P* value
Age	1.02	0.994–1.046	0.134			
Gender						
Female	1					
Male	2.74	1.141–6.59	0.024	2.889	0.823–10.149	0.098
Coinfection						
Without	1					
HBV	0.848	0.242–2.968	0.797			
HAV	0.707	0.043–11.631	0.808			
HAV + HBV	0.707	0.043–11.631	0.808			
Liver cirrhosis						
Without	1					
With	5.576	0.663–46.926	0.114			
ALB	0.777	0.698–0.864	< 0.001	0.843	0.722–0.986	0.032
TBA	1.006	1.002–1.010	0.002	1.002	0.997–1.007	0.376
TB	1.012	1.007–1.016	< 0.001	1.007	1.001–1.013	0.018
ALT	1.0	1.00–1.00	0.841			
AST	1.00	1.00–1.00	0.578			
ALP	1.003	0.998–1.008	0.261			
GGT	1.00	0.998–1.001	0.714			
LDH	1.00	1.000–1.001	0.448			
CHE	0.999	0.999–1.000	< 0.001	1.000	0.999–1.001	0.836
BUN	1.117	0.959–1.301	0.155			
Cr	1.006	0.994–1.018	0.321			
PTA	0.977	0.961–0.993	0.005	1.003	0.980–1.026	0.828
TC	0.654	0.426–1.004	0.052			
TG	1.863	1.013–3.427	0.045	1.328	0.676–2.608	0.410
LDL	0.464	0.272–0.793	0.005	0.930	0.438–1.978	0.851
WBC	1.141	0.912–1.429	0.249			
HB	0.971	0.952–0.991	0.004	0.993	0.964–1.023	0.645
PLT	0.996	0.992–1.001	0.125			
N%	1.047	1.010–1.086	0.012	1.037	0.986–1.091	0.156

## Discussion

Viral hepatitis is a group of diseases characterized by hepatocyte damage caused by a hepatitis virus infection, that mediates inflammation. However, hepatitis viruses can also spread to other tissues and cells; for example, HBV can infect the kidneys and cause hepatitis B related nephropathy, HEV can spread to the central nervous system and cause Guillain–Barre syndrome [37]. Cholecystitis, a common extrahepatic manifestation of liver disease, has been reported more frequently in hepatitis A; however, only a few cases have been reported in HE. First, we retrospectively analyzed 114 patients with acute HE who did not have gallstones or undergo cholecystectomy or gallbladder imaging to determine the exact incidence of cholecystitis. Surprisingly, this study found that 57.89% of patients with sporadic acute HE had signs of acute acalculous cholecystitis, indicating that cholecystitis is very common in acute HE. However, the strains infected in all the PCR-positive cases in this study were confirmed to be genotype 4 by sequencing. Ken Fujioka et al. reported a case of cholecystitis secondary to genotype 1 HEV infection in 2016 [25]. In 2020, ER et al. also reported a case report of overlapping HAV and HEV infection with cholecystitis, but only serological results were available without genotype data [33]. Therefore, whether acalculous cholecystitis is specific to genotype 4 HEV infection or similar manifestations are present in other genotypes requires further investigation.

It has been previously reported that HBV and HAV can cause acalculous cholecystitis. Therefore, we compared the incidence of cholecystitis caused by HEV infection alone and coinfected with HBV and/or HAV. There was no difference in the incidence of cholecystitis between the HEV-alone and HBV superinfection and/or HAV groups. The multivariate analysis showed similar results. These results suggest that acalculous cholecystitis is an inherent phenomenon of acute HE. However, the mechanism of acalculous cholecystitis in patients with hepatitis E remains unclear. Based on previous and the present study, we speculate that there may be some possible mechanisms as follows. Since evidence of HEV replication has been reported in bile duct epithelial cells in animal models [38], so whether gallbladder inflammation is caused by HEV directly or by immune responses against HEV in human needs to be further verified. In addition, when HEV causes inflammation of hepatocytes, the bile secreted by the hepatocytes may contain elevated inflammatory cytokines. And when the bile containing increased inflammatory cytokines flow through the gallbladder, it may stimulate thickening and edema of the gallbladder wall [39]. Furthermore, it has been shown that bacterial translocation can occur in various acute and chronic liver diseases [40], and also bacterial infection is one of the important mechanisms of acalculous cholecystitis. The present study showed that the proportion of patients with spontaneous peritonitis in the presence of cholecystitis was also significantly higher than that in patients without cholecystitis. Therefore, whether bacterial translocation is involved in the development of cholecystitis in patients with hepatitis E requires further investigation.

To obtain more information on the mechanisms and their clinical meaning, we further analyzed the clinical outcomes and biochemical parameters between the two groups of patients with and without cholecystitis. Since bile is synthesized and secreted by hepatocytes and excreted through the biliary tract, we initially wondered whether cholecystitis was due to more severe hepatocyte damage, leading to metabolite changes in bile and cholestasis. However, there was no significant difference between the two groups in the levels of ALT, AST, ALP, GGT, and LDH, which reflect hepatocyte damage and cholestasis. Interestingly, the indicators that reflected the anabolic capacity of the liver, such as ALB, CHE, TB, and PTA were significantly lower than those in the group without cholecystitis. Multivariate analysis showed that ALB and TB were the two major risk factors for the occurrence of acalculous cholecystitis. However, the causality between acalculous cholecystitis and the decline of anabolic function is currently difficult to determine, and further research is needed. Bacterial infections have also been identified as an important cause of acalculous cholecystitis. In the present study, patients with cholecystitis also had a significantly higher incidence of spontaneous peritonitis and neutrophil percentage than those without cholecystitis. In terms of clinical outcomes, there was no significant difference in the incidence of liver failure and mortality between the two groups, but the mean hospital stay in the cholecystitis group was significantly longer than that in the non-cholecystitis group, consistent with the worse anabolic indices in this group, suggesting that gallbladder inflammation may serve as a potential indicator of poor prognosis.

Nevertheless, the present study is retrospective, and the sample size of patients with liver failure and death was small. Therefore, our findings could be biased. In the future, prospective studies with a larger sample size are needed to clarify the value of mechanisms of acalculous cholecystitis as an extrahepatic manifestation of HE.

## Data Availability

The datasets used and/or analysed during the current study are available from the corresponding author on reasonable request.

## Abbreviations

HE: Hepatitis E
HEV: Hepatitis E virus
ALB: Albumin
TBA: Total bile acid
TB: Total bilirubin
DB: Direct bilirubin
ALT: Alanine aminotransferase
AST: Aspartate aminotransferase
ALP: Alkaline phosphatase
GGT: Gamma-glutamyltransferase
LDH: Lactate dehydrogenase
CHE: Cholinesterase
BUN: Blood urea nitrogen
Cr: Creatinine
PTA: Prothrombin activity
TC: Total cholesterol
TG: Triglyceride
LDL: Low density lipoprotein

## References

[1] Teshale EH, Hu DJ (2011). Hepatitis E: epidemiology and prevention. World J Hepatol.

[2] Perez-Gracia MT, Suay-Garcia B, Mateos-Lindemann ML (2017). Hepatitis E and pregnancy: current state. Rev Med Virol.

[3] Purdy MA, Drexler JF, Meng XJ, Norder H, Okamoto H, Van der Poel WHM, Reuter G, de Souza WM, Ulrich RG, Smith DB (2022). ICTV Virus Taxonomy Profile: Hepeviridae 2022. J Gen Virol.

[4] Kamani L, Padhani ZA, Das JK (2021). Hepatitis E: Genotypes, strategies to prevent and manage, and the existing knowledge gaps. JGH Open.

[5] Shafat Z, Ahmed A, Parvez MK, Parveen S (2022). Analysis of codon usage patterns in open reading frame 4 of hepatitis E viruses. Beni Suef Univ J Basic Appl Sci.

[6] Smith DB, Izopet J, Nicot F, Simmonds P, Jameel S, Meng XJ, Norder H, Okamoto H, van der Poel WHM, Reuter G, Purdy MA (2020). Update: proposed reference sequences for subtypes of hepatitis E virus (species Orthohepevirus A). J Gen Virol.

[7] Sridhar S, Yip CC, Wu S, Chew NF, Leung KH, Chan JF, Zhao PS, Chan WM, Poon RW, Tsoi HW (2021). Transmission of rat hepatitis e virus infection to humans in Hong Kong: a clinical and epidemiological analysis. Hepatology.

[8] Pallerla SR, Harms D, Johne R, Todt D, Steinmann E, Schemmerer M, Wenzel JJ, Hofmann J, Shih JWK, Wedemeyer H (2020). Hepatitis E virus infection: circulation, molecular epidemiology, and impact on global health. Pathogens.

[9] Wang B, Meng XJ (2021). Hepatitis E virus: host tropism and zoonotic infection. Curr Opin Microbiol.

[10] Fu Y, Pang L, Dai W, Wu S, Kong J (2022). Advances in the study of acute acalculous cholecystitis: a comprehensive review. Dig Dis.

[11] Treinen C, Lomelin D, Krause C, Goede M, Oleynikov D (2015). Acute acalculous cholecystitis in the critically ill: risk factors and surgical strategies. Langenbecks Arch Surg.

[12] Friberg J, Sönstabö R, Tjick Joe G, Goes E, Osteaux M (1987). Acalculous cholecystitis as a complication of hepatitis. Eur J Radiol.

[13] Velev V, Popov M, Tomov L, Golemanov B (2019). Involvement of the gallbladder in the course of viral hepatitis A in childhood. Trop Doct.

[14] Kaya S, Eskazan AE, Ay N, Baysal B, Bahadir MV, Onur A, Duymus R (2013). Acute acalculous cholecystitis due to viral hepatitis A. Case Rep Infect Dis.

[15] Suresh DR, Srikrishna R, Nanda SK, Annam V, Sunil K, Arjun B (2009). Acalculous gallbladder distension in a young child due to HAV infection: Diagnostic dilemma. Indian J Clin Biochem.

[16] Souza LJ, Braga LC, Rocha Nde S, Tavares RR (2009). Acute acalculous cholecystitis in a teenager with hepatitis a virus infection: a case report. Braz J Infect Dis.

[17] Arroud M, Benmiloud S, Oudghiri B, Afifi MA, Hida M, Bouabdallah Y (2009). Acute acalculous cholecystitis revealing hepatitis A virus infection in children. Saudi J Gastroenterol.

[18] Bouyahia O, Khelifi I, Bouafif F, Mazigh Mrad S, Gharsallah L, Boukthir S (2008). Hepatitis A: a rare cause of acalculous cholecystitis in children. Med Mal Infect.

[19] Başar O, Kisacik B, Bozdogan E, Yolcu OF, Ertugrul I, Köklü S (2005). An unusual cause of acalculous cholecystitis during pregnancy: hepatitis A virus. Dig Dis Sci.

[20] Ciftci AO, Karnak I, Tanyel FC (2001). The association of hepatitis A virus infection, acalculous cholecystitis, and blunt abdominal trauma: a diagnostic challenge. J Pediatr Gastroenterol Nutr.

[21] Unal H, Korkmaz M, Kirbas I, Selcuk H, Yilmaz U (2009). Acute acalculous cholecystitis associated with acute hepatitis B virus infection. Int J Infect Dis.

[22] Omar A, Osman M, Bonnet G, Ghamri N (2016). Acute acalculous cholecystitis caused by Hepatitis C: a rare case report. Int J Surg Case Rep.

[23] Wright WF, Palisoc K, Pinto CN, Lease JA, Baghli S (2020). Hepatitis C virus-associated acalculous cholecystitis and review of the literature. Clin Med Res.

[24] Gaisinskaya P, Sugerik S, Gebara CM (2022). Acalculous cholecystitis secondary to hepatitis C infection. Cureus.

[25] Fujioka K, Nishimura T, Seki M, Kinoshita M, Mishima N, Irimajiri S, Yamato M (2016). Genotype 1 hepatitis E virus infection with acute acalculous cholecystitis as an extrahepatic symptom: a case report. Trop Med Health.

[26] Deniel C, Coton T, Brardjanian S, Guisset M, Nicand E, Simon F (2011). Acute pancreatitis: a rare complication of acute hepatitis E. J Clin Virol.

[27] Wang L, Gao F, Lin G, Yuan Y, Huang Y, Hao H, Zhuang H, Wang L (2018). Association of hepatitis E virus infection and myasthenia gravis: a pilot study. J Hepatol.

[28] Zheng X, Yu L, Xu Q, Gu S, Tang L (2018). Guillain-Barre syndrome caused by hepatitis E infection: case report and literature review. BMC Infect Dis.

[29] Brehm TT, Mazaheri O, Horvatits T, Lutgehetmann M, SchulzeZurWiesch J, Lohse AW, Polywka S, Pischke S (2021). Lower levels of transaminases but higher levels of serum creatinine in patients with acute hepatitis E in comparison to patients with hepatitis A. Pathogens.

[30] Leaf RK, O'Brien KL, Leaf DE, Drews RE (2017). Autoimmune hemolytic anemia in a young man with acute hepatitis E infection. Am J Hematol.

[31] Shah SA, Lal A, Idrees M, Hussain A, Jeet C, Malik FA, Iqbal Z, Rehman H (2012). Hepatitis E virus-associated aplastic anaemia: the first case of its kind. J Clin Virol.

[32] Ibrahim AS, Alkhal A, Jacob J, Ghadban W, Almarri A (2009). Hepatitis E in Qatar imported by expatriate workers from Nepal: epidemiological characteristics and clinical manifestations. J Med Virol.

[33] Piza Palacios L, Espinoza-Rios J (2020). Hepatitis A and hepatitis E virus co-infection with right pleural effusion, ascites and acute acalculous cholecystitis. A case report. Rev Gastroenterol Peru.

[34] Suda T, Iguchi R, Ishiyama T, Kanefuji T, Hoshi T, Abe S, Morita S, Yagi K (2021). A superinfection of salmonella typhi and hepatitis E virus causes biphasic acute hepatitis. Intern Med.

[35] Huffman JL, Schenker S (2010). Acute acalculous cholecystitis: a review. Clin Gastroenterol Hepatol.

[36] Barie PS, Eachempati SR (2010). Acute acalculous cholecystitis. Gastroenterol Clin North Am.

[37] Zhou X, Huang F, Xu L, Lin Z, de Vrij FMS, Ayo-Martin AC, van der Kroeg M, Zhao M, Yin Y, Wang W (2017). Hepatitis E virus infects neurons and brains. J Infect Dis.

[38] Kawai HF, Koji T, Iida F, Kaneko S, Kobayashi K, Nakane PK (1999). Shift of hepatitis E virus RNA from hepatocytes to biliary epithelial cells during acute infection of rhesus monkey. J Viral Hepat.

[39] Kim MY, Baik SK, Choi YJ, Park DH, Kim HS, Lee DK, Kwon SO (2003). Endoscopic sonographic evaluation of the thickened gallbladder wall in patients with acute hepatitis. J Clin Ultrasound.

[40] Chopyk DM, Grakoui A (2020). Contribution of the intestinal microbiome and gut barrier to hepatic disorders. Gastroenterology.

